# Supplementary material for: Resolving the full spectrum of human genome variation using Linked-Reads
Source: Genome Res. 2019 Apr;29(4):635–45. doi: 10.1101/gr.234443.118 (PMC6442396; doi:10.1101/gr.234443.118)
Supplement: Supplemental Material [file supp_gr.234443.118_Supplemental_longranger-2.2.zip › longranger-2.2.tar.bz2!/lr2.2-public/lib/stan/cmdstan/stan/doc/api/html/matrix_2meta_2value__type_8hpp_source.html]

Stan: src/stan/math/matrix/meta/value\_type.hpp Source File


|  |  |
| --- | --- |
|  | Stan  2.5.0  probability, sampling & optimization |


- Main Page
- Related Pages
- Namespaces
- Classes
- Files

- File List
- File Members

All Classes Namespaces Files Functions Variables Typedefs Enumerations Enumerator Friends Macros Pages

- src
- stan
- math
- matrix
- meta

value\_type.hpp

Go to the documentation of this file.

1 #ifndef STAN\_\_MATH\_\_MATRIX\_\_META\_\_VALUE\_TYPE\_HPP

2 #define STAN\_\_MATH\_\_MATRIX\_\_META\_\_VALUE\_TYPE\_HPP

3

4 #include <stan/math/meta/value\_type.hpp>

5 #include <Eigen/Core>

6

7 namespace stan {

8

9  namespace math {

10

19  template <typename T, int R, int C>

20  struct value\_type<Eigen::Matrix<T, R, C> > {

21  typedef typename Eigen::Matrix<T,R,C>::Scalar type;

22  };

23

24

25  }

26

27 }

28

29

30 #endif

value\_type.hpp

stan::math::value\_type< Eigen::Matrix< T, R, C > >::type

Eigen::Matrix< T, R, C >::Scalar type

**Definition:** value\_type.hpp:21

stan::math::value\_type

Primary template class for metaprogram to compute the type of values stored in a container.

**Definition:** value\_type.hpp:21


---

[ Stan Home Page ]

*© 2011–2014,
Stan Development Team.*
